# The Effect of Foaming Agents on the Thermal Behavior of Aluminum Precursors

**DOI:** 10.3390/ma17030710

**Published:** 2024-02-01

**Authors:** Tomislav Rodinger, Danko Ćorić, Jaroslav Kováčik

**Affiliations:** 1 Department of Materials, Faculty of Mechanical Engineering and Naval Architecture, University of Zagreb, Ivana Lučića 5, 10000 Zagreb, Croatia; 2 Institute of Materials and Machine Mechanics, Slovak Academy of Sciences, Dúbravská cesta 9/6319, 84513 Bratislava, Slovakia

**Keywords:** aluminum foam, powder metallurgy, foaming agent, foaming temperature

## Abstract

Various foaming agents can be used to achieve foaming of the precursors obtained by using the powder metallurgy method. However, the thermal behavior of pure aluminum precursors with different foaming agents has been studied very little in recent times. For the production of aluminum foams with closed cells, 1 wt.% of calcium carbonate (CaCO_3_), titanium hydride (TiH_2_), heat-treated TiH_2_ and zirconium hydride (ZrH_2_) were used. The foaming capability of the compacted precursors was investigated at temperatures 700, 720 and 750 °C. CaCO_3_ and TiH_2_ showed the best foamability at all considered temperatures, while ZrH_2_ achieved relatively good foaming only at the highest temperature, 750 °C. Due to their low onset temperature of the decomposition compared to the melting point of the unalloyed aluminum, in hydride-based foaming agents the drainage occurred at the bottom part of the foam samples. Among the investigated foaming agents, precursors with heat-treated TiH_2_ had the worst foaming properties, while CaCO_3_ showed the best foamability without the occurrence of drainage.

## 1. Introduction

Cellular materials, such as metal foams, recently have been attracting considerable attention due to their specific structure and unique properties [1,2,3,4]. Because of good energy absorption, they can be used for crash-boxes in road and rail vehicles or to reduce vibrations in various machines [1,5,6]. If biocompatible materials are used to produce foams, they can also be used in biomedical engineering for the implants or prostheses. In this case, human tissue can grow into the foam cells, resulting in a better connection between the implant and the bone [1,6,7]. A promising functional application of cellular materials is in thermal engineering, where metal foams can find various applications, from thermal energy storage systems to heat sinks [8,9,10].

As their name suggests, foams are not as present monolithic (non-porous) metals, but also consist of a large number of cells (pores) bounded between metallic walls. Metal foams differ according to the size, distribution, and the type of their cells, which can be open or closed [11]. The different cell types are obtained by different production methods. The most common techniques for the closed-cell foams are the foaming of molten metal and the foaming of compacted metal powders [12,13]. In the case of molten metal foaming, various gases can be used to produce foams. Foaming agents in the form of powders can be used for metal precursors (green compacts) foaming and for melt foaming. These agents decompose into solid and gaseous phases at elevated temperatures [14]. At temperatures at which metal is in a liquid or semi-solid state, the gas remains trapped inside the metal, and with fast enough solidification, a metallic foam is formed. In addition to the standard methods of producing cellular metals, with the increase in additively manufactured metallic materials, some examinations were also focused on additively produced porous structures. Unlike other methods, this enables the production of periodic cellular lattice structures, but they focus on materials with open cells [15,16,17,18].

When producing metal foams with closed cells by the powder metallurgy method, the process consists of several phases [19]. Previously characterized metal and foaming agent powders must be mixed to obtain a homogeneous mixture with evenly distributed foaming agent, so that these particles cause uniform cell formation throughout the material. After mixing, the powders must be pressed into green compacts. According to Paulin et al. [20], to achieve the best foaming characteristics, the relative density of the precursor should be above 98%, although it is possible to achieve foaming even with lower density values, but with a worse foamability. If the precursor material is porous, the gas escapes through the porous channels outside of the material, so the remaining amount of gas trapped inside the metal is not high enough to trigger foaming. After powders compacting, the next step is to put the precursor into the mold and place it in a heated furnace, where the foaming process follows. The furnace should be heated to a sufficient temperature for metal melting and to initiate the decomposition of the foaming agent. The molds can have different shapes and sizes, depending on the requested foam form. Based on the mold size and the degree of porosity, the required mass of the precursor should be determined.

The foaming agents have different onset temperatures for decomposition. The most commonly used agent, titanium hydride (TiH_2_) [21,22,23], begins to decompose at around 450 °C [24,25,26]. It is desirable that this temperature is close to the melting point of the base material of the foam (metal of cell walls). Several different methods of TiH_2_ processing have been developed to delay its decomposition temperature, such as heat treatment of TiH_2_ powder to form a surface layer of titanium oxides (TiO, TiO_2_, Ti_3_O) [27]. Precursors with TiH_2_ agent can also be heat treated (annealed). In this case, the foams have a more isotropic pore structure and fewer defects, which is due to the more uniform foaming behavior [28]. Due to the significantly lower price, foaming with calcium carbonate (CaCO_3_) has recently been increasingly investigated [29,30,31,32]. In addition to the financial benefits, the decomposition temperature, which starts at around 660 °C [29,33,34], is also an advantage for unalloyed aluminum that melts at 660 °C. In contrast to TiH_2_, which forms relatively large cells, CaCO_3_ forms a finer morphological structure [35]. This is because foaming starts at the later stage when the temperature is close to the aluminum melting point. Also, carbon dioxide (CO_2_) released from CaCO_3_ in contact with aluminum forms an aluminum oxide (Al_2_O_3_) layer on the cell walls, which prevents them from collapsing during cooling [31,36]. In addition to these two foaming agents, numerous other agents were also investigated, mostly based on hydrides and carbonates [37,38,39,40].

Depending on the melting temperature of the base metal and the decomposition temperature of the foaming agent, it is necessary to choose the appropriate foaming temperature. If the selected temperature is too low, not enough gas will be released from the foaming agent, or the precursor will not be in a softened state to allow the growth and foam development. If the furnace temperature is too high, the matrix metal melts and the melt flows to the bottom of the mold, and after solidification there is no porous structure in the lower part, only monolithic (compact) metal. In addition, the gas can escape more easily from the melted precursor and the foaming is weaker. With prolonged holding of the precursor at a temperature that is too high, the cells start to merge and form larger ones, which leads to weaker mechanical properties at those locations.

Most of the literature sources focus on the foaming of aluminum alloys with TiH_2_ agent, so the aim of this study was to investigate the influence of other agents (CaCO_3_, ZrH_2_ and original and heat-treated TiH_2_) on the foaming of pure (unalloyed) aluminum. To examine the thermal behavior of precursors from compacted aluminum powder and foaming agent, experiments were carried out at different temperatures: 700, 720 and 750 °C. After foaming, the shape of the samples, with regard to the mold volume as well as the cell morphology on the cross-section, was analyzed. The pore features, such as pore size and distribution, were examined and it was observed if there was melt drainage at the bottom of the mold.

## 2. Materials and Methods

With the aim of aluminum precursor production by using the powder metallurgy method, aluminum metal powder A1050 (99.7 wt.% purity, Metallpulver24, Sankt Augustin, Germany) and various foaming agents were used. Four types of precursors were prepared, each with a different foaming agent, as follows: CaCO_3_ (98.5 wt.% purity, Gram-mol, Zagreb, Croatia), ZrH_2_ (99.0 wt.% purity, type F, Chemetall GmbH, Frankfurt, Germany) and original (non-heat-treated) and heat-treated (HT) TiH_2_ (98.8 wt.% purity, Chemetall GmbH, Frankfurt, Germany). The chemical composition of powder mixtures is shown in Table 1.

The quantity of foaming agent in each of these precursors was 1 wt.%. For the heat treatment process, the TiH_2_ powder was annealed in air atmosphere at a temperature of 450 °C for 60 min. Foaming agents decompose in a certain temperature range and form a solid (S) and a gaseous (G) phase according to the following equations:

CaCO_3(S)_ → CaO_(S)_ + CO_2(G)_, (1)

ZrH_2(S)_ → Zr_(S)_ + H_2(G)_, (2)

TiH_2(S)_ → Ti_(S)_ + H_2(G)_. (3)

The gaseous phase forms foam cells if it remains trapped in the matrix metal, in this case aluminum.

Aluminum and foaming agent particle size analysis was carried out for each used powder by using the wet laser diffraction method on an Analysette 22 (Fritsch, Idar-Oberstein, Germany) laser particle sizer. Then, aluminum powder and a foaming agent were accurately weighed to obtain four different mixtures, each containing 1 wt.% of the following agents: CaCO_3_, TiH_2_, heat-treated TiH_2_ and ZrH_2_. Then, 150 g of powders were mixed in air atmosphere in a Turbula T2F (WAB, Basel, Switzerland) mixer for 60 min at 72 rpm with complex rotation to achieve a good dispersion of the foaming agent in the aluminum powder matrix.

The homogeneous mixtures were cold isostatically pressed (CIP) (IMMM SAS, Bratislava, Slovakia) in a hermetically sealed rubber mold at a pressure of 150 MPa and room temperature. As the samples were not compact enough after CIP, they were directly extruded (DE) (IMMM SAS, Bratislava, Slovakia) at 400 °C to achieve the highest possible density. The extrusion speed was 0.5 mm/s, with an extrusion ratio of 9:1. After extrusion, obtained precursors had the final shape of rectangular strips.

These precursors were cut into smaller pieces to fit into a cube-shaped graphite mold with internal dimensions of 40 mm × 40 mm × 40 mm. The same quantity of different precursors was used for all samples, approximately 62 g, which should result in 65% porosity:

(4) $$porosity=\left(1-\frac{\rho_{foam}}{\rho_{Al}}\right)\cdot 100\%$$

where *ρ*_foam_ is foam density, calculated as a foam mass and volume ratio, while *ρ*_Al_ is aluminum density.

Foaming was carried out in an electric furnace (VEB Elektro, Bad Frankenhausen, Germany) at different temperatures (700, 720 and 750 °C) to observe the foaming properties of examined agents at various temperatures. The design of the experiment is presented in Table 2.

Closed molds filled with the precursor were placed in a preheated furnace. The molds were kept in the furnace for about 10 min until the molten aluminum began to flow out through the hole at the top of the mold, indicating the end of the foaming process. After cooling, the sample was removed from the mold and the quality of the foaming success was validated according to the achieved sample form. The samples were then cut in a direction perpendicular to the original precursor’s position in the mold. The cell size and their distribution were analyzed, as well as whether all precursor pieces were fully foamed. Samples were checked for the eventual occurrence of melt drainage. To determine the number and size of cells, cross-sections were processed in an image analyzing software, ImageJ 1.54 g (NIH, Bethesda, MD, USA).

## 3. Results and Discussion

The results of powders particle size analysis, as the mean value of two measurements and standard deviation, are shown in Table 3. CaCO_3_ powders have a bimodal distribution, while the other powders have a single modal distribution. Although the analysis showed that CaCO_3_ powders have the smallest particles, they tend to agglomerate into larger clusters very easily during the mixing process.

**Figure 1:**
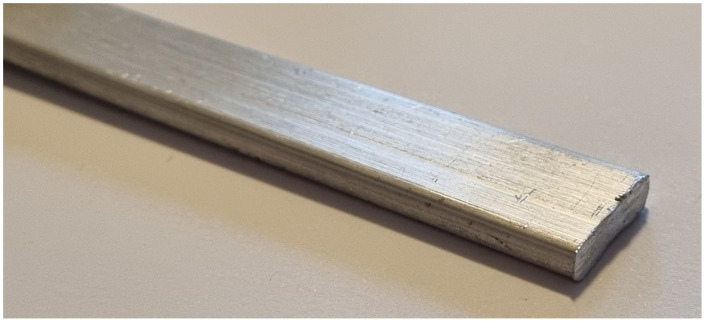
Produced precursor after CIP and DE compaction processes.

After preparing a homogeneous mixture of aluminum powder and foaming agent in a content of 1 wt.%, cold isostatic pressing was carried out (according to Section 2). The CIP green compacts had a relative density (the ratio of measured and theoretical density) of about 85%, which is not enough for a successful foaming process. If such material was placed in a furnace chamber, after the decomposition of the foaming agent into the solid and gaseous phase, all the gas would escape through the porous channels of the precursor without initiating the foaming process. To obtain a much higher compactness of the powders, the pressed samples were subsequently directly extruded at 400 °C, which increased their relative densities close to the theoretical values. The extruded precursors had a rectangular cross-section area, with dimensions of 5 mm × 20 mm, Figure 1.

The relative density of the extruded precursors (*ρ*_prec_) was calculated as the ratio of their density measured by the Archimedes’ principle (*ρ*_measured_) in distilled water on the analytical balance, type AS 220/X (Radwag, Radom, Poland), and the theoretically achievable density (*ρ*_theo_):

(5) $$\rho_{prec}=\frac{\rho_{measured}}{\rho_{theo}}.$$

The theoretical density includes the mass percents of the individual powders and their densities:

(6) $$\rho_{theo}=0.99\cdot \rho \left(Al\right)+0.01\cdot \rho (foaming\;agent).$$

The following density values were used for the calculation of theoretical precursor densities: *ρ*(Al) = 2.7 g/cm^3^, *ρ*(CaCO_3_) = 2.71 g/cm^3^, *ρ*(TiH_2_) = 3.75 g/cm^3^ and *ρ*(ZrH_2_) = 5.60 g/cm^3^ [41]. The measured, theoretical and relative precursor densities are listed in Table 4. The values of measured densities are average values of two measurements with standard deviation indicated.

As shown in Table 2, relative densities above 99% were obtained for all samples using the combination of CIP and extrusion powder compaction methods.

Samples with hydride-based precursors were foamed at 700, 720 and 750 °C, while carbonate-based precursors were foamed only at 720 °C and 750 °C (Table 2). The lowest temperature was not examined as the CaCO_3_ just starts to decompose into calcium oxide (CaO) and carbon dioxide (CO_2_) at around 660 °C [29] which is already very close to 700 °C.

At a temperature of 700 °C, foaming of hydride-based precursors was not successful, except for original TiH_2_. Since not enough heat reached the upper precursor pieces, some of them barely started to foam. This is consistent with the observations of Nosko et al. [42] in which the placement of precursors in the bottom part of the mold in multiple layers led to inhomogeneous foaming from the bottom precursor pieces, with delayed pore nucleation in the upper precursor pieces. This is the result of the different thermal conductivity of the precursor and the foam: the bottom precursor pieces start to foam, but as the foam develops, the thermal conductivity of the foam decreases. As a result, the heat flow to the upper precursor pieces decreases considerably and their foaming is significantly delayed. Only the precursor with the non-heat-treated TiH_2_ foaming agent had relatively good foamability, but even this precursor did not fill the entire mold.

**Figure 2:**
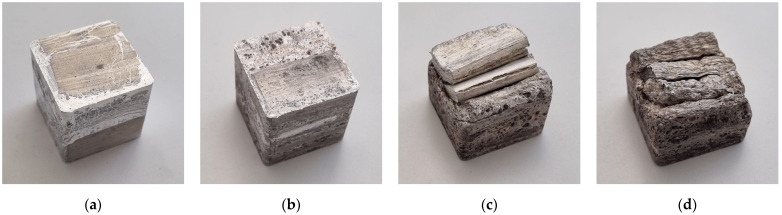
Samples foamed at 720 °C with different foaming agents: (**a**) CaCO_3_; (**b**) TiH_2_; (**c**) heat-treated TiH_2_; (**d**) ZrH_2_.

At a higher temperature (720 °C), both non-heat-treated TiH_2_ precursor and precursor with CaCO_3_ showed good foaming properties and filled the whole mold, as can be seen in Figure 2. It was again confirmed that the delayed foaming occurs in the top row of precursor pieces for heat-treated TiH_2_ and ZrH_2_. At an even higher foaming temperature (750 °C), CaCO_3_ and non-heat-treated TiH_2_ precursors have good foaming properties. The precursor with ZrH_2_ foaming agent showed significantly better foamability than at lower temperatures and only included a smaller number of imperfections on the sample surface.

Some of the samples were held in the furnace for too long while waiting for the molten aluminum to start to flow out through the hole in the mold, which did not happen due to unsuccessful foaming. Due to the extended time the metal remained in a molten state, the foam simply began to collapse. This can be seen in Figure 2d, which shows a rather wrinkled top of the foamed sample with ZrH_2_ foaming agent.

**Figure 3:**
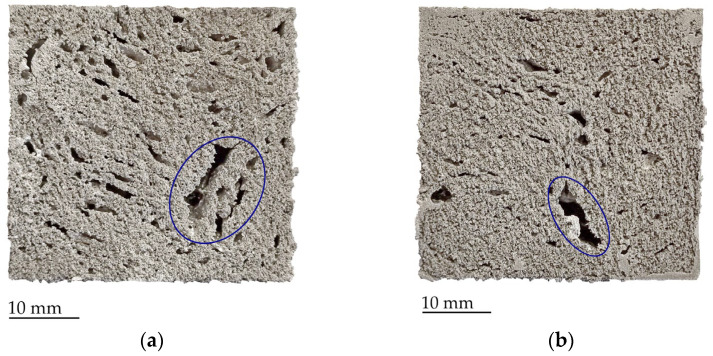
Cross-sections of samples with CaCO_3_ foaming agent foamed at: (**a**) 720 °C; (**b**) 750 °C, with the voids between unconnected precursors marked blue.

**Figure 4:**
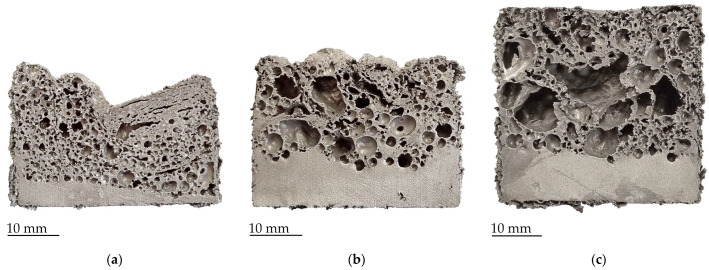
Cross-sections of samples with ZrH_2_ foaming agent foamed at (**a**) 700 °C; (**b**) 720 °C; (**c**) 750 °C.

**Figure 5:**
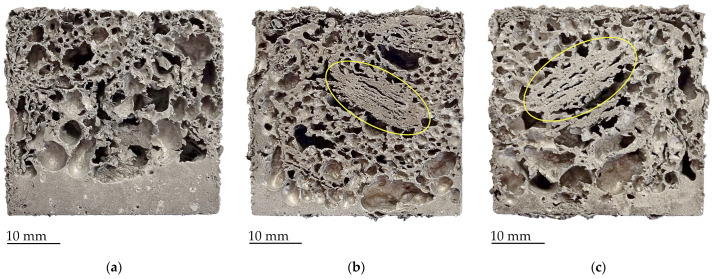
Cross-sections of samples with TiH_2_ foaming agent foamed at: (**a**) 700 °C; (**b**) 720 °C; (**c**) 750 °C; not fully foamed precursors marked yellow.

**Figure 6:**
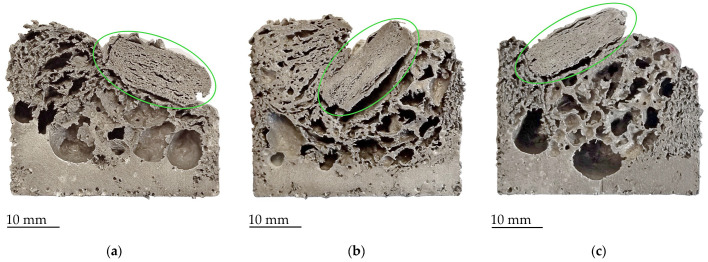
Cross-sections of samples with heat-treated TiH_2_ foaming agent foamed at (**a**) 700 °C; (**b**) 720 °C; (**c**) 750 °C; not fully foamed precursors marked green.

In the analysis of samples cross-section (Figure 3, Figure 4, Figure 5 and Figure 6), it is noticeable that in almost all cases where hydride-based foaming agents were used, the molten aluminum can be observed in the lower part. This was not observed when the carbonate-based agent was used. As expected, on the cross-sections of the samples with CaCO_3_ precursors (Figure 3), it can be observed that the foams have the smallest cells, although there are also a few larger voids (marked blue on Figure 3). These cavities are not the result of the formation of large cells due to the premature release of CO_2_ from the foaming agent, but probably the result of the non-joining of the material of the two neighboring precursor pieces, i.e., the cavity that remained between the precursors.

In ZrH_2_ samples (Figure 4), more and more drainage occurs with the increase in the temperature. As hydride-based foaming agents start the decomposition into hydrogen and the solid phase at a temperature that is relatively low compared to the aluminum melting point, smaller cells formed at the very early stage start to merge and grow into larger ones [11]. Contrary to hydride-based foaming agents, the carbonate-based agent starts to decompose at a temperature that roughly corresponds to the aluminum melting point. At the considered temperatures of 720 °C and 750 °C, the foaming has only just started, and the cells have not yet started to merge into larger ones.

Samples with TiH_2_ foaming agent showed very good foamability, with only minor deviations from the mold shape. At the samples cross-section (Figure 5), some drainage can be seen, especially at 700 °C, and slightly less drainage at higher foaming temperatures. Some precursor pieces did not join well together and were still not even fully foamed at temperatures 720 °C and 750 °C (marked yellow on Figure 5), since they did not receive enough heat. The cells have a very large variation in size and distribution.

The precursors with heat-treated TiH_2_ powder showed the worst foaming properties overall (Figure 6). This is the result of a partial release of hydrogen from the TiH_2_ powder during annealing. Thus, the amount of hydrogen was probably not enough to allow successful foaming. As with the two previous sample groups with hydride-based foaming agents, the foam cells vary greatly in size, ranging from very small to huge cells.

Since the precursor pieces were placed on top of each other, the lowest precursors have the largest contact surface with the walls of the mold, and they heat up to foaming temperature faster than other pieces. In cases where hydride-based foaming agents were used, this results in the formation of drainage in the lower part of the mold, while some precursor pieces in the upper part of the mold did not yet begin to foam, or foaming was only in the initial phase (marked green on Figure 6a–c).

Gergely and Clyne defined in their work [43] that for production of foams with minimal density gradients, i.e., to minimize drainage during foam production, attention should be paid to rapid foaming (to minimize the period during which porosity is low) and on the cell walls’ stabilization (to prevent the cell walls from breaking up and thus to maintain finer cells). In this case, CaCO_3_ serves both purposes—it improves rapid foaming by CO_2_ evolution around the melting temperature of aluminum and promotes foam stabilization. In contact with aluminum, CO_2_ forms an Al_2_O_3_ layer and contributes to better stabilization of the cell walls. Hydrides do not improve the stabilization of the foam at all. Therefore, for pure aluminum, the only option is to use a hydride with the best hydrogen evolution.

Up to the considered foaming temperatures, TiH_2_ releases almost all the hydrogen stored in the Ti structure: Borchers et al. [44] observed the thermal desorption spectrum (TDS) of the interrupted decomposition of TiH_2_. The decomposition starts around 400 °C. The TDS spectrum consists of a narrow main peak (*T*_max_ = 544 °C) as well as low- and high-temperature shoulders (*T*_l_ = 470 °C and *T*_h_ = 647 °C, respectively). At 750 °C, almost all hydrogen is released from TiH_2_, leaving only 0.04 H/Ti, i.e., hydrogen atom per Ti at 757 °C [44]. This is probably the reason why TiH_2_ is the best hydride-based choice for foaming of aluminum in an electric furnace.

Ma et al. [45] observed similar TDS spectrum for ZrH_2_ and found one main peak (around 827 °C), three low-temperature peaks (470, 590 and 710 °C) and one high-temperature shoulder (867 °C). The evolution of hydrogen from ZrH_2_ starts at about 380 °C and at 980 °C there is only 0.03 H/Zr. In the case of this study, ZrH_2_ at 750 °C still contains a considerable amount of hydrogen; 1.35 H/Zr, according to Ma et al. [45]. The initial value at room temperature is 2 H/Zr, which means that only 33% of the total hydrogen stored in the foaming agent is released up to 750 °C. This low amount of released hydrogen leads to slow foaming and insufficient filling of the mold with aluminum foam in this study.

Banhart and Ritter [46] concluded that during TiH_2_ powder annealing in the air atmosphere, the hydrogen loss is less than 10% of the total hydrogen and that this loss is acceptable given the known benefits of heat treatment. However, our results for pure aluminum foams prepared with heat-treated TiH_2_ powder (air, 450 °C, 60 min) show that this is not true for unalloyed aluminum. Due to the heat treatment, i.e., the loss of a certain amount of hydrogen, it was not possible in the experiments to fill the mold with foam when the weight of the precursor corresponded to a relative porosity of about 65%.

Table 5 shows the number of cells on the samples cross-section and their mean and maximum size, as well as standard deviation. It shows also the foamability of used precursors.

The samples with CaCO_3_ foaming agent have a multiple times higher number of cells compared to the hydride-based foaming agents. In addition, their mean size and standard deviation, as well as the maximum pore size, are significantly smaller. For Al-CaCO_3_ samples, the largest cells are not created by the gas released from the foaming agent, but they are the gaps between the two neighboring precursors that did not come into full contact during the foaming process. The largest cells consist mostly of several cells interconnected by smaller channels or by breaking the thin cell-walls.

## 4. Conclusions

The powder metallurgy method was used to produce closed-cell pure aluminum foams. The compacted aluminum precursors were prepared with 1 wt.% of different foaming agent powders: CaCO_3_, TiH_2_, heat-treated TiH_2_ and ZrH_2_. By cold isostatic pressing and extrusion at 400 °C, the relative densities of the green compacts higher than 99% were achieved. The foaming was carried out in the range from 700 to 750 °C to observe the effect of temperature and foaming agent powder on foamability.

The following conclusions can be drawn from the conducted research:

(i) At a temperature of 700 °C, foaming of all precursors, except original (non-annealed) TiH_2_, was unsuccessful due to delayed pore nucleation. Too low of a temperature in the mold prevented foaming of the upper precursor pieces located near the top of the mold. Furthermore, at a temperature of 700 °C, the amount of gas released from the ZrH_2_ agent is too small for complete foaming.
(ii) Calcium carbonate is the most effective foaming agent in the case of pure aluminum as it promotes rapid foaming through the development of carbon dioxide, which also helps to stabilize the cell walls. Thus, they create foams with a very large number of small cells due to the later onset of gas release from the foaming agent without drainage of the melt in the lower part of the sample.
(iii) Unlike CaCO_3_, hydrides do not contribute to foam stabilization. The choice of hydride-based foaming agent for pure aluminum is more crucial. TiH_2_ proves to be the preferred option as it releases the most hydrogen stored in its structure up to 750 °C. The samples with the original TiH_2_ powder showed very good foaming properties. There was little drainage at the bottom of the samples and the foam cells were larger compared to the cells formed by the CaCO_3_ agent. But their size varied greatly due to the unstable structure because some cells start to merge and form larger ones.
(iv) Heat-treated TiH_2_ agent has low foaming properties. This is the result of hydrogen loss during annealing, which is usually considered as the best approach for aluminum alloy foams, but not in the case of studied pure aluminum.
(v) Samples with ZrH_2_ foaming agent have shown low foamability at lower temperatures. At 750 °C, the foaming was relatively good due to the higher hydrogen volume released, which makes the cells grow, but large melt drainage appears at the bottom of the sample.

With the goal of further understanding of aluminum foams, research that follows will be focused on evaluating their mechanical properties with the aim of determining a clear correlation between structure and properties.

## Figures and Tables

**Table 1:** Chemical composition of powder mixtures.

Mixture	Chemical Composition
Al-CaCO_3_	99 wt.% Al + 1 wt.% CaCO_3_
Al-TiH_2_	99 wt.% Al + 1 wt.% TiH_2_
Al-TiH_2_ (HT)	99 wt.% Al + 1 wt.% TiH_2_ (HT)
Al-ZrH_2_	99 wt.% Al + 1 wt.% ZrH_2_

**Table 2:** Design of experiment.

Precursor	Foaming Temperatures, °C		
Al-CaCO_3_	–	720	750
Al-TiH_2_	700	720	750
Al-TiH_2_ (HT)	700	720	750
Al-ZrH_2_	700	720	750

**Table 3:** Particle size distribution of the used powders (*d*_50_ and *d*_90_ indicate that 50% or 90% of the analyzed powder particles have smaller size than specified).

Material	*d*_50_, μm	*d*_90_, μm
Aluminum	60.32 ± 0.57	102.48 ± 0.26
CaCO_3_	2.26 ± 0.20	11.67 ± 0.80
TiH_2_	17.34 ± 0.11	37.52 ± 0.01
TiH_2_ (HT)	16.29 ± 0.18	37.74 ± 0.46
ZrH_2_	6.33 ± 0.04	17.46 ± 0.59

**Table 4:** Measured, theoretical and relative densities of extruded precursors.

Precursor	Measured Density, g/cm³	Theoretical Density, g/cm³	Relative Density, %
Al-CaCO_3_	2.6861 ± 0.0021	2.7001	99.48
Al-TiH_2_	2.7013 ± 0.0057	2.7105	99.66
Al-TiH_2_ (HT)	2.7023 ± 0.0039	2.7105	99.70
Al-ZrH_2_	2.7119 ± 0.0033	2.7290	99.37

**Table 5:** Number of cells and their size for different samples.

Sample	Foaming Temperature, °C	Number of Cells	Cell Size, mm^2^			Foamability
			Mean Size	Standard Deviation	Maximum Size	
Al-CaCO_3_	720	18,458	0.02315	0.34739	31.76052	+
	750	12,451	0.01567	0.32500	33.29073	+
	700	762	1.16649	11.42545	245.42725	+
Al-TiH_2_	720	611	1.21161	9.81946	201.5518	+
	750	476	1.71642	11.23842	181.45033	+
	700	359	1.37276	12.02811	168.69567	−
Al-TiH_2_ (HT)	720	526	0.69720	7.13230	118.32997	−
	750	807	0.62731	5.89777	141.79737	−
	700	535	0.64446	2.14417	23.79973	−
Al-ZrH_2_	720	200	1.58381	7.34641	92.04191	−
	750	350	2.25070	15.94923	218.48624	+

## Data Availability

Data are contained within the article.
